# Nonlinear radiative Maxwell nanofluid flow in a Darcy–Forchheimer permeable media over a stretching cylinder with chemical reaction and bioconvection

**DOI:** 10.1038/s41598-021-88947-5

**Published:** 2021-04-30

**Authors:** Chunyan Liu, Muhammad Usman Khan, Muhammad Ramzan, Yu-Ming Chu, Seifedine Kadry, M. Y. Malik, Ronnason Chinram

**Affiliations:** 1 School of Science, Beijing University of Civil Engineering and Architecture, Beijing, 100044 People’s Republic of China; 2 Beijing Key Laboratory of Functional Materials for Building Structure and Environment Remediation, Beijing University of Civil Engineering and Architecture, Beijing, 100044 People’s Republic of China; 3 Department of Computer Science, Bahria University, Islamabad, 44000 Pakistan; 4 Department of Mathematics, Huzhou University, Huzhou, 313000 People’s Republic of China; 5 Hunan Provincial Key Laboratory of Mathematical Modeling and Analysis in Engineering, Changsha University of Science and Technology, Changsha, 410114 People’s Republic of China; 6 Faculty of Applied Computing and Technology, Noroff University College, Kristiansand, Norway; 7 Department of Mathematics, College of Sciences, King Khalid University, Abha, 61413 Kingdom of Saudi Arabia; 8 Division of Computational Science, Faculty of Science, Prince of Songkla University, Hat Yai, Songkhla, 90110 Thailand

**Keywords:** Software, Mechanical engineering

## Abstract

Studies accentuating nanomaterials suspensions and flow traits in the view of their applications are the focus of the present study. Especially, the usage of such materials in biomedical rheological models has achieved great importance. The nanofluids’ role is essential in the cooling of small electronic gizmos like microchips and akin devices. Having such exciting and practical applications of nanofluids our goal is to scrutinize the Maxwell MHD nanofluid flow over an extended cylinder with nonlinear thermal radiation amalgamated with chemical reaction in a Darcy–Forchheimer spongy media. The presence of gyrotactic microorganisms is engaged to stabilize the nanoparticles in the fluid. The partial slip condition is considered at the boundary of the stretching cylinder. The Buongiorno nanofluid model is betrothed with impacts of the Brownian motion and thermophoresis. The analysis of entropy generation is also added to the problem. The highly nonlinear system is tackled numerically is addressed by the bvp4c built-in function of the MATLAB procedure. The outcomes of the prominent parameters versus embroiled profiles are portrayed and conversed deeming their physical significance. It is perceived that fluid temperature is augmented for large estimates of the radiation and Darcy parameters. Moreover, it is noticed that the magnetic and wall roughness parameters lower the fluid velocity. To corroborate the presented results, a comparison of the current study with a previously published paper is also executed. An outstanding correlation in this regard is attained.

## Introduction

Nanofluid, an arising field of engineering, has caught the eye of numerous researchers who were observing the ways to improve the efficiency of cooling measures in industries. Nanofluids are used to improve rates of heat transfer in an assortment of applications including nuclear reactors, transportation industry, mechanical cooling applications, heat exchangers, micro-electromechanical systems, fiber, and granular insulation, chemical catalytic reactors, packed blood flow in the cardiovascular system engaging the Navier–Stokes equation. Advanced thermal features of the nanofluid are imperative in many fields like pharmaceutical, air-conditioning, micromanufacturing, microelectronics, power generation, thermal therapy for cancer surgery, transportation, chemical, and metallurgical engineering fields, etc. Due to the significant advancement in aerodynamics automotive, there is great importance in breaking down systems by direct heat dissipation. Many investigators have recently added some work to promote solar cells with high digestion of solar radiation. As Choi and Eastman^1^ found that the incorporation of nanoparticles to the base liquids significantly enhances their thermal efficiency. The rising demand for highly efficient cooling devices encourages Koo and Kleinstreuer^2^ to study the steady laminar nanofluid flow in micro heat sinks. It is noticed that very low nanoparticle concentration in nanofluids results in a higher thermal conductivity that exhibits a remarkable state of nanofluids^3,4^. Bilal et. al^5^ scrutinized the numerical study of unsteady Maxwell flow of nanofluid influenced by the magnetic field, melting heat, and the Fourier and Fick laws. This investigation reveals that the liquid temperature is dropped for versus melting heat and unsteadiness parameters. The flow of 3D non-radiative Maxwell nanofluid with thermal and solutal stratification with chemical reaction is analytically studied by Tlili et. al^6^. Here, the noticeable outcome of the model is that the fluid concentration and temperature are declined for solutal and thermal stratifications respectively. Farooq et al.^7^ analytically conversed the Maxwell nanofluid flow over an exponentially extended surface. Various researchers revealed the numerous aspects of the Williamson nano liquid^7–22^.

The term Darcy–Forchheimer comes from the law of Darcy which interprets the liquid flow along a spongy channel. This law was originated and dependent upon the consequences of analysis on the water flow across the beds of sand. Movements in the spongy medium in which inertial effects are prominent come with the variations of Reynolds numbers. Therefore, this introductory term is adding up to the Darcy equation and is referred to as the Darcy–Forchheimer term. This term represents the non-linear behavior of the flow data versus pressure difference. With wide utilization of grain stockpiling, petroleum technology, frameworks of groundwater and oil assets, this Darcy law is of immense importance in the field of Fluid Mechanics. In places where the porous medium has larger flow rates due to non-uniformity, such as near the wall, Darcy's law is not applicable. The substance with stomata is named as a permeable medium. It includes an application of large numbers such that oil manufacturing, liquid flow in catalytic vessels, and reservoirs, etc. The suggestion of the fluid flow passes a porous surface was first given by Darcy^23^ in 1856. However, this idea couldn't be so famous inferable from its restrictions of lower porosity and smaller speed. Afterward, Forchheimer^24^ amended the equation of momentum by adding the square velocity condition into the Darcian velocity to convey the undeniable lack. Muskat^25^ later call it the "Forchheimer term" which is true of the high Reynolds number. Pal and Mondal^26^ addressed the Darcy–Forchheimer model over permeable media past the linearly expanded region and assume that concentration distribution is diminishing function of the electric field parameter. The movement of the hydromagnetic nano liquid past the Darcy–Forchheimer media forum effect on the boundary condition of second order is mathematically evaluated by Ganesh et al.^27^. Alshomrani et al.^28^ explained the 3D Darcy–Forchheimer law with carbon nanotubes and homogeneous heterogeneous reactions. The viscous nanofluid with Darcy–Forchheimer effect over a curved area is analyzed by Saif et al.^29^. Seth et al.^30^ examined mathematically the movement of carbon nanotubes over a porous Darcy–Forchheimer media in a rotating frame and many therein^31–41^.

In numerous processes including dispersion of nutrients in nerves, condensation in mixtures, and thermal insulation, mass transfer plays a vital role. One live example of transfer of mass may be seen in the living matter processes like respiration and sweating. A good number of studies may be quoted where chemical reactions play a vital role in mass transfer procedures.

Recently, Mahmood^42^ explored the nanofluid flow with an amalgamation of the CNT’s of both types and the engine oil over a stretched surface with the impact of the activation energy merged with the chemical reaction. The problem is solved numerically and with a surface response statistical technique. It is inferred from this model that the surface drag coefficient is negatively sensitive concerning the magnetic parameter. The numerical solution of the nanofluid flow involving the CNT’s and water over an extended/contracting sheet with quartic autocatalysis chemical reaction and Thompson and Troian slip boundary conditions is discussed by Ramzan et al.^43^. The results exposed that the fluid concentration is enhanced for quartic autocatalysis chemical reaction. Khan et al.^44^ numerically tackled the Carreau nanofluid flow over an extended surface in a Homann stagnation region with chemical reaction and modified Fourier law using shooting scheme. The study divulges that the fluid velocity hinders owing to the Hartmann number and the porosity parameter. In the perspective of its clarity, the remarkable work of current researchers, see few studies^45–56^.

In thermodynamics, entropy is an essential concept. One of the most powerful methods for investigating the efficiency of thermal systems is entropy generation analysis. The idea of irreversibility is inextricably related to the concept of entropy. All have an instinctive understanding of irreversibility. For example, by playing a video game in both forward and reverse, we can merely explain the irreversibility phenomenon by using backward order. There are numerous forward procedures of daily life that cannot be undone, such as pouring water into a bottle, egg unscrambling, unconstrained expansion of fluids, plastic deformation, gas uprising from the chimney, etc. In this perspective, Yusuf et al.^57^ explored the entropy generation in a Maxwell fluid flow over an inclined extended surface in a non-Darcian spongy media with thermal radiation. An analytical solution of the erected mathematical model is attained. The major result of the presented model is that the rate of the entropy generation is boosted for the local inertial coefficient parameter. In a recent study, Adesanya et al.^58^ performed the entropy generation appraisal for a couple stress fluid film flow on an inclined heated surface with viscous dissipation impacts. In this analysis, it is comprehended that the fluid temperature and velocity show opposing tendency versus the couple stress parameter. Furthermore, many investigators have worked on entropy generation analysis on a wide number of geometries that may found in^58–65^.

The studies deliberated above reveal that abundant literature discussing the nanofluid flow of an extended cylinder is available under the influence of varied impacts. Nevertheless, comparatively less literature can be witnessed that discusses the flow of Maxwell nanofluid over an extended cylinder. But so far one has discussed the Maxwell nanofluid flow past an extended cylinder with thermal radiation, chemical reaction, and gyrotactic microorganisms with partial slip in a Darcy–Forchheimer spongy medium. The problem is solved numerically, and pertinent graphs are plotted versus the involved profiles with logical descriptions. Table 1 illustrates the originality/uniqueness of the stated fluid mathematical model by assessing it with the available researches.

**Table 1:** A literature analysis for the individuality of the stated model.

Authors	Buongiorno model	Maxwell nanofluid flow over a cylinder	Darcy–Forchheimer impact	Nonlinear thermal radiation	Bioconve-ction	Chemical reaction
Islam et al.^15^	√	√	×	×	×	×
Ahmed et al.^16^	√	√	×	√	×	×
Hayat et al.^66^	√	√	×	×	×	√
Raju et al.^67^	√	√	×	×	×	×
Present	√	√	√	√	√	√

(√) means said effect present, and ( ×) signifies the impact is absent.

## Mathematical modeling

**Figure 1:**
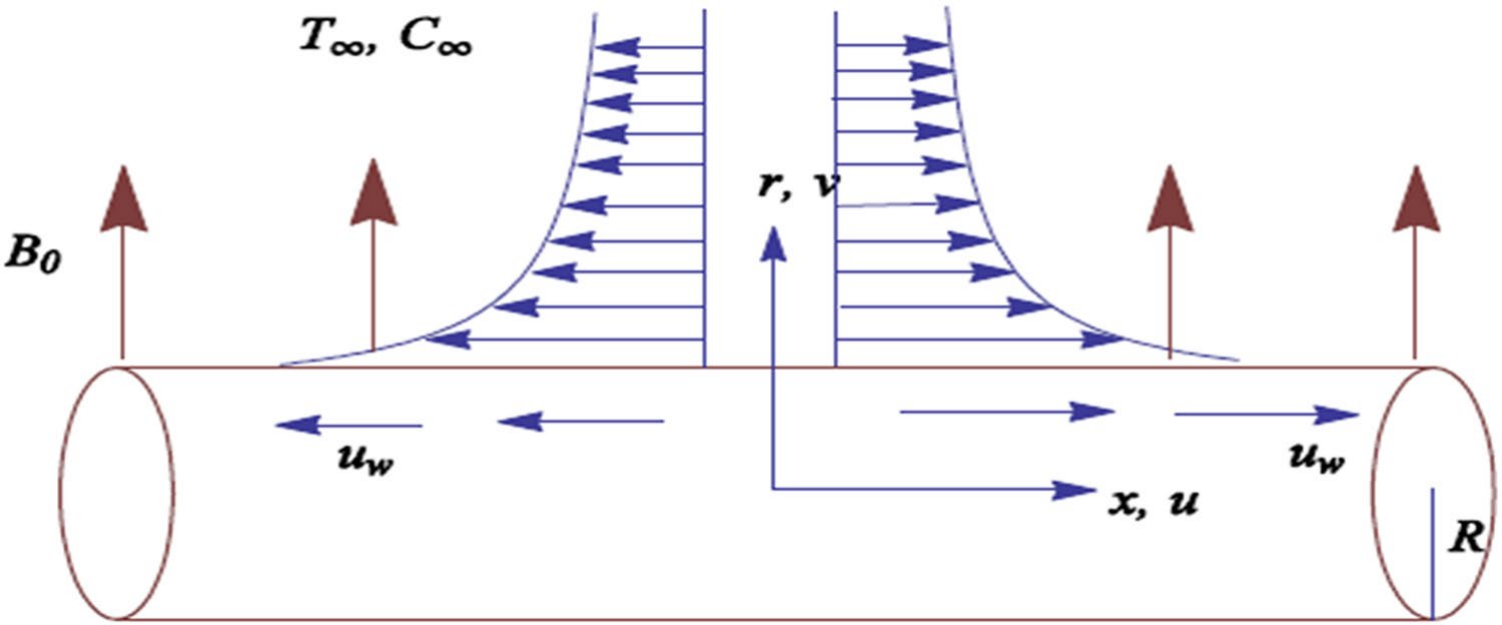
The geometry of the flow.

We examine an incompressible flow outside a cylinder having radius R and the constant temperature $T_{w}$. As the axial direction of a cylinder along the *x-*axis while radial direction along *r-*axis. A stretching surface of the cylinder has velocity $u_{w} = u_{0} (\frac{x}{l})$, where $l$ is the characteristic length and $u_{0}$ shows the reference velocity. The flow situation induced by a magnetic field of intensity $B_{0}$ is displayed in Fig. 1.

The resulting boundary layer equations defining the depicted scenario are given as^66^:

1 $$\frac{\partial }{\partial x}\left( {ru} \right) + \frac{1}{r}\frac{\partial }{\partial r}\left( {rv} \right) = 0,$$

2 $$u\frac{\partial u}{{\partial x}} + v\frac{\partial u}{{\partial r}} = \nu \left( {\frac{{\partial^{2} u}}{{\partial r^{2} }} + \frac{1}{r}\frac{\partial u}{{\partial r}}} \right) + \lambda^{*} \left[ {u^{2} \frac{{\partial^{2} u}}{{\partial x^{2} }} + 2uv\frac{{\partial^{2} u}}{\partial x\partial r} + v^{2} \frac{{\partial^{2} u}}{{\partial r^{2} }}} \right] - \frac{{\sigma_{1} }}{\rho }B_{0}^{2} u - \frac{\nu }{{k_{2} }}u - Fu^{2} ,$$

3 $$u\frac{\partial T}{{\partial x}} + v\frac{\partial T}{{\partial r}} = \frac{k}{{\rho c_{p} }}\left( {\frac{1}{r}\frac{\partial T}{{\partial r}} + \frac{{\partial^{2} T}}{{\partial r^{2} }}} \right) + \tau \left[ {D_{B} \frac{\partial T}{{\partial r}}\frac{\partial C}{{\partial r}} + \frac{{D_{T} }}{{T_{\infty } }}\left( {\frac{\partial T}{{\partial r}}} \right)^{2} } \right] - \frac{1}{{\rho c_{p} }}\frac{{\partial q_{r} }}{\partial r},$$

4 $$u\frac{\partial C}{{\partial x}} + v\frac{\partial C}{{\partial r}} = D_{B} \left( {\frac{{\partial^{2} C}}{{\partial r^{2} }} + \frac{1}{r}\frac{\partial C}{{\partial r}}} \right) + \frac{{D_{T} }}{{T_{\infty } }}\left( {\frac{{\partial^{2} T}}{{\partial r^{2} }} + \frac{1}{r}\frac{\partial T}{{\partial r}}} \right) - \lambda_{1} \left( {C - C_{\infty } } \right)^{n} ,$$

5 $$u\frac{\partial N}{{\partial x}} + v\frac{\partial N}{{\partial r}} = D_{n} \left( {\frac{1}{r}\frac{\partial N}{{\partial r}} + \frac{{\partial^{2} N}}{{\partial r^{2} }}} \right) - \frac{{\lambda w_{c} }}{{C_{w} - C_{\infty } }}\left[ {\frac{\partial C}{{\partial r}}\frac{\partial N}{{\partial r}} + N\frac{{\partial^{2} C}}{{\partial r^{2} }}} \right].$$

With boundary conditions^66^:

6 $$\begin{gathered} u = L_{1} v\frac{\partial u}{{\partial r}}\left| {_{r = R} { + }\,\,\,{\text{u}}_{w} {,}\,\,\, \, v} \right. = 0, \hfill \\ T = L_{2} \frac{\partial T}{{\partial r}}\left| {_{r = R} } \right.{ + }T_{w} {, }C = L_{3} \frac{\partial C}{{\partial r}} + C_{w} . \hfill \\ N = L_{4} \frac{\partial N}{{\partial r}}\left| {_{r = R} { + }N_{w} \, } \right.{\text{at}}\,\,\,r = R, \hfill \\ u = 0,{\text{ N}} = N_{\infty } ,\,\,\,C = C_{\infty } ,\,\,\,\,\, \, T = T_{\infty } ,{\text{ as}}\,\,\,{\text{at}}\,\,r = \infty , \hfill \\ \end{gathered}$$

The radiative heat flux $q_{r}$ is given by:

7 $$q_{r} = \left( { - \frac{{4\sigma^{*} }}{{3k^{*} }}} \right)\frac{{\partial T^{4} }}{\partial r} = \left( { - \frac{{16\sigma^{*} }}{{3k^{*} }}} \right)T^{3} \frac{\partial T}{{\partial r}}.$$

The following transformation is used to obtain the non-dimensional structure of the above-mentioned flow model^66^:

8 $$\begin{gathered} \eta = \frac{{r^{2} - R^{2} }}{2R}\left( {\frac{{u_{0} }}{l\nu }} \right)^{\frac{1}{2}} , \, \psi = \sqrt {\left( {\frac{{\nu u_{0} }}{l}} \right)} xRf\left( \eta \right), \, \theta \left( \eta \right) = \frac{{T - T_{\infty } }}{{T_{w} - T_{\infty } }}, \hfill \\ \xi \left( \eta \right) = \frac{{N - N_{\infty } }}{{N_{w} - N_{\infty } }}, \, \phi \left( \eta \right) = \frac{{C - C_{\infty } }}{{C_{w} - C_{\infty } }}, \, u = \frac{{u_{0} }}{l}xf^{^{\prime}} \left( \eta \right), \, v = - \frac{1}{r}\sqrt {\frac{{\nu u_{0} }}{l}} Rf\left( \eta \right). \hfill \\ \end{gathered}$$

Equation (1) trivially fulfilled and Eqs. (2–6) are as follows:

9 $$\begin{gathered} \left( {1 + 2M\eta } \right)f^{\prime\prime\prime} + ff^{\prime\prime} - f^{{\prime}{2}} + 2Mf^{\prime\prime} - \beta \left( {f^{2} f^{\prime\prime\prime} - 2ff^{\prime\prime}} \right) \hfill \\ - K^{2} f^{\prime} - \lambda f^{\prime} - Frf^{{\prime}{2}} = 0, \hfill \\ \end{gathered}$$

10 $$\begin{gathered} \frac{1}{\Pr }\left[ {\left( {1 + 2M\eta } \right)\theta ^{\prime\prime} + 2M\theta ^{\prime}} \right] + f\theta ^{\prime} + Nb\left( {1 + 2M\eta } \right)\theta ^{\prime}\phi ^{\prime} + Nt\left( {1 + 2M\eta } \right)\theta ^{{\prime}{2}} \hfill \\ + \frac{1}{\Pr }\frac{4}{3}Rd\left[ \begin{gathered} 3\left( {\theta_{w} - 1} \right)\left[ {1 + \left( {\theta_{w} - 1} \right)\theta } \right]^{2} \left( {1 + 2M\eta } \right)\theta ^{{\prime}{2}} + \left[ {1 + \left( {\theta_{w} - 1} \right)\theta } \right]^{3} M\theta ^{\prime} \hfill \\ + \left[ {1 + \left( {\theta_{w} - 1} \right)\theta } \right]^{3} \left( {1 + 2M\eta } \right)\theta ^{\prime\prime} \hfill \\ \end{gathered} \right] = 0, \hfill \\ \end{gathered}$$

11 $$\left( {1 + 2M\eta } \right)\phi ^{\prime\prime} + 2M\phi ^{\prime} + Scf\phi ^{\prime} + \frac{Nt}{{Nb}}\left[ {\left( {1 + 2M\eta } \right)\theta ^{\prime\prime} + 2M\theta ^{\prime}} \right] - Sc\gamma \phi^{n} = 0,$$

12 $$\begin{gathered} \left( {1 + 2M\eta } \right)\xi^{^{\prime\prime}} + Lb\Pr f\xi^{^{\prime}} + 2M\xi^{^{\prime}} \hfill \\ - Pe\left( \begin{gathered} \sigma M\phi^{^{\prime}} + M\xi \phi^{^{\prime}} + \left( {1 + 2M\eta } \right)\xi^{^{\prime}} \phi^{^{\prime}} \hfill \\ + \sigma \left( {1 + 2M\eta } \right)\phi^{^{\prime\prime}} + \left( {1 + 2M\eta } \right)\xi \phi^{^{\prime\prime}} \hfill \\ \end{gathered} \right) = 0. \hfill \\ \end{gathered}$$

13 $$\begin{gathered} f\left( 0 \right) = 0, \, f^{^{\prime}} \left( 0 \right) = B_{1} f^{^{\prime\prime}} \left( 0 \right) + 1, \, \theta \left( 0 \right) = B_{2} \theta^{^{\prime}} \left( 0 \right) + 1, \hfill \\ \phi \left( 0 \right) = B_{3} \phi^{^{\prime}} \left( 0 \right) + 1, \, \xi \left( 0 \right) = B_{4} \left( 0 \right)\xi ^{\prime}(0) + 1, \hfill \\ f^{^{\prime}} = 0, \, \theta = 0, \, \phi = 0, \, \xi = 0. \hfill \\ \end{gathered}$$

Particular dimensionless parameters emerging in the above equations are portrayed as:

14 $$\begin{gathered} M = \left( {\frac{l\nu }{{u_{0} R^{2} }}} \right)^{\frac{1}{2}} ,\quad K = \left( {\frac{{\sigma_{1} B_{0}^{2} l}}{{\rho u_{0} }}} \right)^{\frac{1}{2}} ,\quad B_{1} = L_{1} \left( {\frac{{u_{0} \nu }}{l}} \right)^{\frac{1}{2}} ,\quad B_{2} = L_{2} \left( {\frac{{u_{0} }}{l\nu }} \right)^{\frac{1}{2}} ,\quad B_{3} = L_{3} \left( {\frac{{u_{0} }}{l\nu }} \right)^{\frac{1}{2}} , \hfill \\ \theta_{w} = \frac{{T_{w} }}{{T_{\infty } }},\quad \gamma = \frac{{\lambda_{1} l}}{{u_{0} }},\quad Nb = \frac{{\tau D_{B} \left( {C_{w} - C_{\infty } } \right)}}{\nu },\quad Nt = \frac{{\tau D_{T} \left( {T_{w} - T_{\infty } } \right)}}{{\nu T_{\infty } }},\quad \beta = \frac{{\lambda^{*} u_{0} }}{l}, \hfill \\ \lambda = \frac{\nu l}{{k_{2} u_{0} }},\quad Fr = \frac{{c_{b} }}{{\sqrt {k_{2} } }},\quad Rd = \left( {\frac{{4\sigma^{*} T_{\infty }^{3} }}{{kk^{*} }}} \right),\quad Sc = \frac{\nu }{{D_{B} }},\quad Pe = \frac{{\lambda w_{c} }}{{D_{n} }}, \hfill \\ Lb = \frac{\alpha }{{D_{n} }},\quad \Pr = \frac{\nu }{\alpha },\quad \sigma = \frac{{N_{\infty } }}{{N_{w} - N_{\infty } }}, \hfill \\ \end{gathered}$$

The dimensional form of drag force coefficients, rate of mass transfer, rate of heat transfer, and Motile microorganisms are appended as below:

15 $$C_{{f_{x} }} = \frac{{\mu \left( {\frac{\partial u}{{\partial r}} + \frac{\partial v}{{\partial x}}} \right)_{r = R} }}{{\rho u_{w}^{2} }},{\text{ Sh}}_{x} = \frac{{xj_{w} }}{{D_{m} \left( {C_{w} - C_{\infty } } \right)}},{\text{ Nu}}_{x} = \frac{{xq_{w} }}{{k\left( {T_{w} - T_{\infty } } \right)}},{\text{ Nn}}_{x} { = }\frac{{xq_{n} }}{{D_{n} \Delta N}}{. }$$

With

16 $$q_{w} = - k\left( {\frac{\partial T}{{\partial r}}} \right)_{r = R} + \left( {q_{r} } \right)_{w} , \, j_{w} = - D_{m} \left( {\frac{\partial C}{{\partial r}}} \right)_{r = R} , \, q_{n} = - D_{n} \left( {\frac{\partial N}{{\partial r}}} \right)_{r = a} ,$$

The Drag force coefficient, mass transfer rate, Motile microorganism, and rate of heat in the dimensionless form are supplemented below:

17 $$\begin{gathered} {\text{Re}}_{x}^{\frac{1}{2}} C_{{f_{x} }} = f^{\prime\prime}\left( 0 \right), \, {\text{Re}}_{x}^{{ - \frac{1}{2}}} Sh_{x} = \, - \phi^{^{\prime}} \left( 0 \right){,} \hfill \\ {\text{Re}}_{x}^{{ - \frac{1}{2}}} Nu_{x} = - \left( {1 + \frac{4}{3}Rd\left( {1 + \left( {\theta_{w} - 1} \right)\theta \left( 0 \right)} \right)} \right)^{3} ,\theta^{^{\prime}} \left( 0 \right),{\text{ Nn}}_{x} = - \xi^{^{\prime}} \left( 0 \right){,}\,\,\,\, \hfill \\ \end{gathered}$$

## Rate of entropy generation (EG)

The volumetric equation is represented as:

18 $$\begin{gathered} S_{G} = \frac{k}{{T_{\infty }^{2} }}\left( {\frac{\partial T}{{\partial r}} + \left( {\frac{{16\sigma T^{3} }}{{3k^{*} }}} \right)\left( {\frac{\partial T}{{\partial r}}} \right)^{2} } \right) + \frac{\mu }{{T_{\infty } }}\left( {\frac{\partial u}{{\partial r}}} \right)^{2} + \frac{{\sigma B_{0}^{2} u^{2} }}{{T_{\infty } \rho }} + \frac{Rd}{{C_{\infty } }}\left( {\frac{\partial C}{{\partial r}}} \right)^{2} + \frac{Rd}{{T_{\infty } }}\left( {\frac{\partial T}{{\partial r}}} \right)\left( {\frac{\partial C}{{\partial r}}} \right) \hfill \\ + \frac{Rd}{{N_{\infty } }}\left( {\frac{\partial N}{{\partial r}}} \right)^{2} + \frac{Rd}{{C_{\infty } }}\left( {\frac{\partial N}{{\partial r}}} \right)\left( {\frac{\partial C}{{\partial r}}} \right). \hfill \\ \end{gathered}$$

The characteristics EG is framed as:

19 $$S^{\prime\prime\prime} = \frac{{\nabla T^{2} k}}{{T_{\infty }^{2} l^{2} }}$$

The entropy generation $N_{G}$ is given as the quotient of the $S_{G}$ and $S^{\prime\prime\prime}$, i.e.,

20 $$N_{G} = \left( {\frac{{S_{G} }}{{S^{\prime\prime\prime}}}} \right)$$

In dimensionless form:

21 $$\begin{aligned} N_{G} & = \left( {\frac{{S_{G} }}{{S^{\prime\prime\prime}}}} \right) = \left( {1 + 2M\eta } \right)\alpha_{1} {\text{Re}} \theta^{^{\prime}2} + \left( {1 + 2M\eta } \right)Br{\text{Re}} f^{^{\prime\prime}2} + MBr{\text{Re}} f^{^{\prime}2} \\ & \quad + \left( {1 + 2M\eta } \right)L{\text{Re}} \theta^{^{\prime}} \phi^{^{\prime}} + \left( {1 + 2M\eta } \right)L_{5} {\text{Re}} \frac{{\alpha_{3} }}{{\alpha_{1} }}\xi^{^{\prime}2} + \left( {1 + 2M\eta } \right)L_{5} {\text{Re}} \frac{{\alpha_{2} }}{{\alpha_{1} }}\xi^{^{\prime}} \phi^{^{\prime}} . \\ \end{aligned}$$

The parameters used in Eq. (22) are defined as:

22 $$\begin{gathered} \alpha_{1} = \frac{{T_{w} - T_{\infty } }}{{T_{\infty } }},\quad \alpha_{2} = \frac{{C_{w} - C_{\infty } }}{{C_{\infty } }},\quad \alpha_{3} = \frac{{N_{w} - N_{\infty } }}{{N_{\infty } }},\quad L = \left( {\frac{{Rd\left( {C_{w} - C_{\infty } } \right)}}{k}} \right) \hfill \\ L_{5} = \left( {\frac{{Rd\left( {N_{w} - N_{\infty } } \right)}}{k}} \right),\quad Br = \left( {\frac{{\mu_{0} a^{2} x^{2} }}{k\Delta T}} \right),\quad N_{G} = \left( {\frac{{S_{G} T_{\infty } \nu }}{k\Delta Ta}} \right),\quad {\text{Re}} = \frac{{U_{0} l^{2} }}{\nu }. \hfill \\ \end{gathered}$$

## Numerical procedure

For the nonlinear arrangement of equations and boundary conditions (9)–(13) the finite difference MATLAB bvp4c procedure is applied which is solid at 4th order and the grid size of 0.01 is viewed as acknowledged 10^–6^. The numerical plan requires the change of higher-order differential equations into one-order differential equations.

23 $$\begin{gathered} y_{1} = f,y_{2} = f^{\prime},y_{3} = f^{\prime\prime},yy_{1} = f^{\prime\prime\prime},y_{4} = \theta ,y_{5} = \theta^{\prime},yy_{2} = \theta^{\prime\prime}, \hfill \\ y_{6} = \phi ,y_{7} = \phi^{\prime},yy_{3} = \phi^{\prime\prime},y_{8} = \xi ,y_{9} = \xi^{\prime},yy_{4} = \xi^{\prime\prime}, \hfill \\ \end{gathered}$$

24 $$\begin{aligned} yy_{1} & = \left( {\frac{{\left( { - y_{1} y_{3} + y_{2}^{2} - 2My_{3} + \beta \left( {y_{1} y_{3} - 2y_{1} y_{3} } \right)} \right) + K^{2} y_{2} + \lambda y_{1} + Fry_{2} y_{2} }}{1 + 2M\eta } - } \right); \\ & \left( { - 2\gamma y_{5} - \Pr y_{1} y_{5} - \Pr \left( {1 + 2M\eta } \right)\left( {N_{b} y_{5} y_{7} + N_{t} y_{5}^{2} } \right)} \right) \\ \end{aligned}$$

25 $$yy_{2} = \frac{{ - \frac{4}{3}Rd\left[ {3\left[ {\theta_{w} - 1} \right]\left( {1 + \left[ {\theta_{w} - 1} \right]y_{4} } \right)^{2} \left( {1 + 2M\eta } \right)y_{5}^{2} + \left( {1 + y_{4} \left[ {\theta_{w} - 1} \right]} \right)^{3} My_{5} } \right]}}{{\left( {1 + 2M\eta } \right) + \frac{4}{3}Rd\left( {1 + \left[ {\theta_{w} - 1} \right]y_{4} } \right)^{3} \left( {1 + 2M\eta } \right)}};$$

26 $$yy_{3} = \left( {\frac{{ - 2My_{7} - Scy_{1} y_{7} - \frac{{N_{t} }}{{N_{b} }}\left( {2My_{5} + (1 + 2M\eta yy_{2} } \right) + ScMy_{6}^{n} }}{(1 + 2\gamma \eta )}} \right);$$

27 $$yy_{4} = \frac{{\left( { - 2My_{9} - Lb\Pr y_{1} y_{9} } \right) + Pe\left( \begin{gathered} \left( {1 + 2M\eta } \right)y_{9} y_{6} + My_{8} y_{7} + \sigma My_{7} \hfill \\ \sigma \left( {1 + 2M\eta } \right)y_{8} yy_{3} + \left( {1 + 2M\eta } \right)y_{8} yy_{3} \hfill \\ \end{gathered} \right)}}{(1 + 2M\eta )};$$

28 $$\begin{gathered} y_{2} (0) - 1;y_{2} (0) - 1 - B_{1} y_{3} (0);y_{4} (0) - 1 - B_{2} y_{5} (0);y_{6} (0) - 1 - B_{3} y_{7} (0); \hfill \\ y_{8} (0) - 1 - B_{4} y_{9} (0);y_{2} (\infty );y_{4} (\infty );y_{6} (\infty );y_{8} (\infty ); \hfill \\ \end{gathered}$$

## Results with discussion

**Figure 2:**
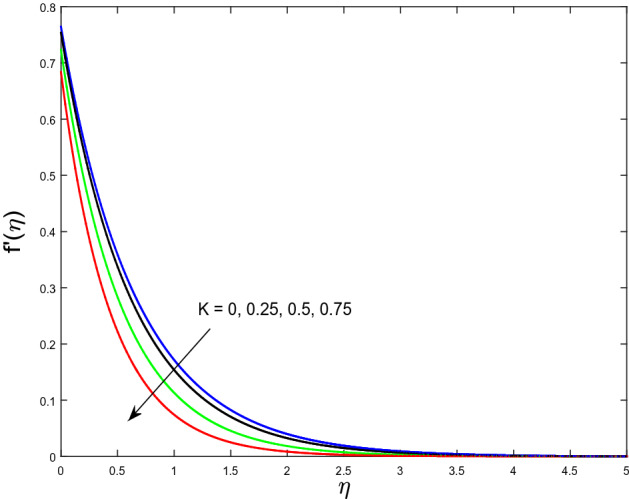
Plot of $f^{^{\prime}}$ for $K$.

**Figure 3:**
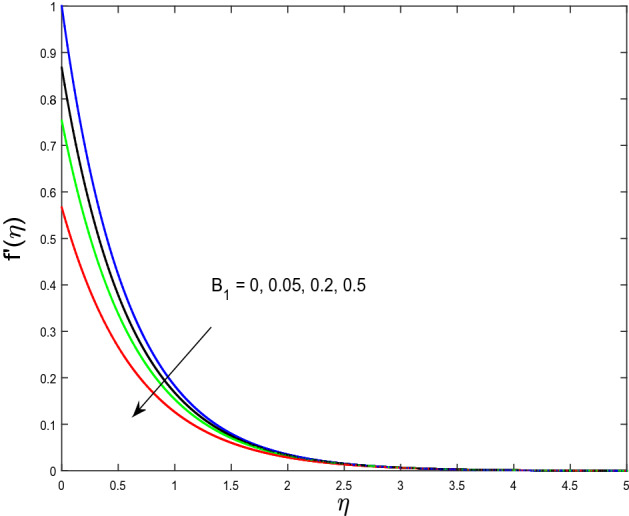
Plot of $f^{^{\prime}}$ for $B_{1}$.

**Figure 4:**
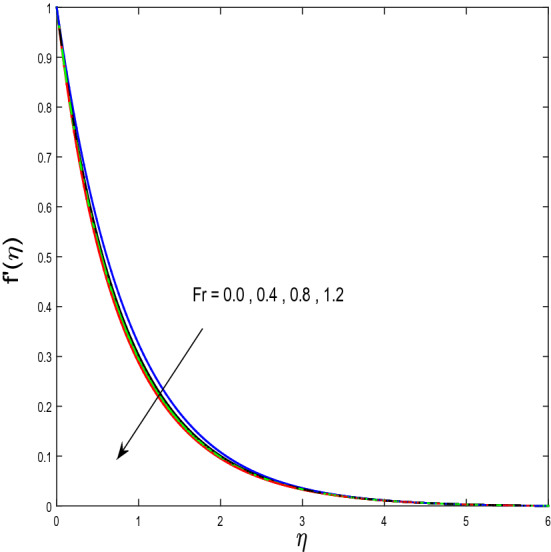
Plot of $f^{^{\prime}}$ for $Fr$.

**Figure 5:**
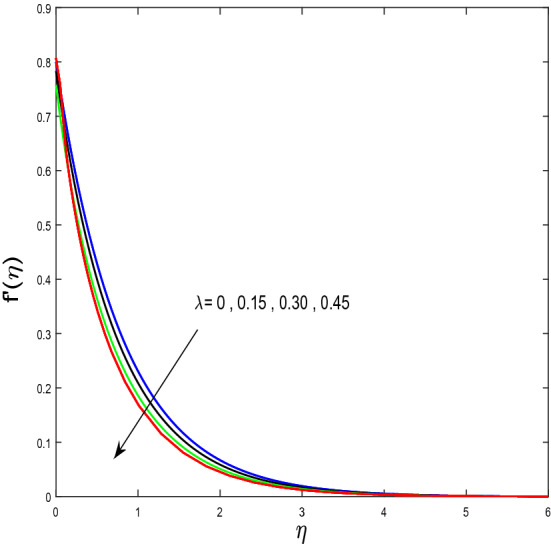
Plot of $f^{^{\prime}}$ for $\lambda$.

**Figure 6:**
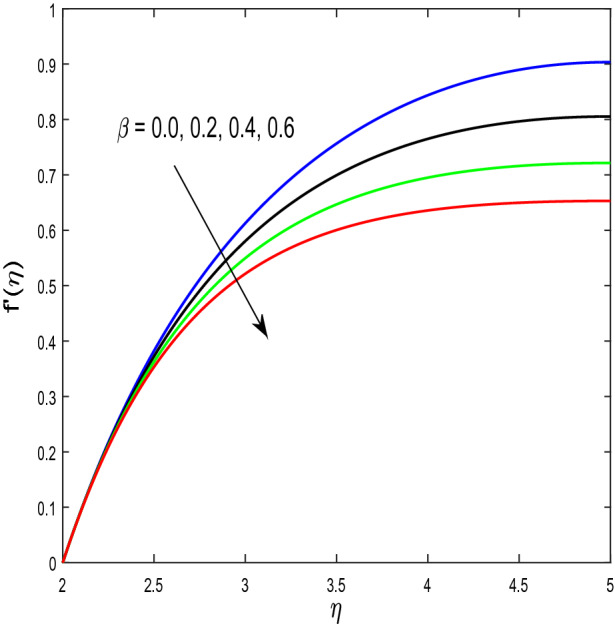
Plot of $f^{^{\prime}}$ for $\beta$.

**Figure 7:**
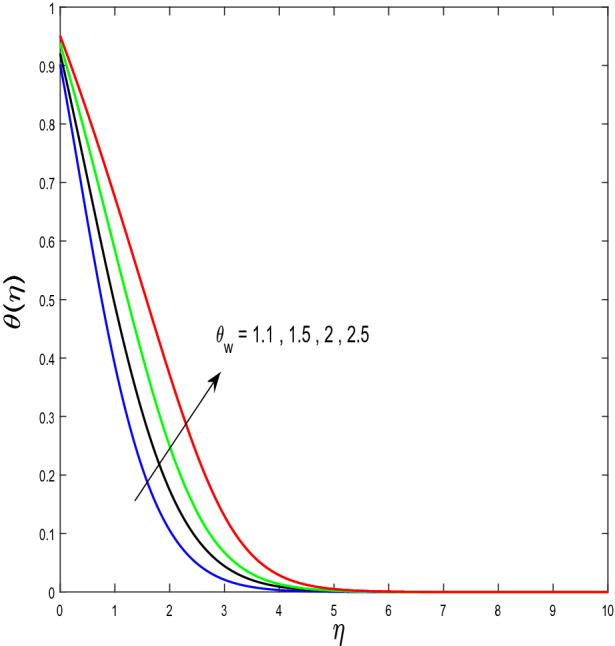
Plot of $\theta$ for $\theta_{w}$.

**Figure 8:**
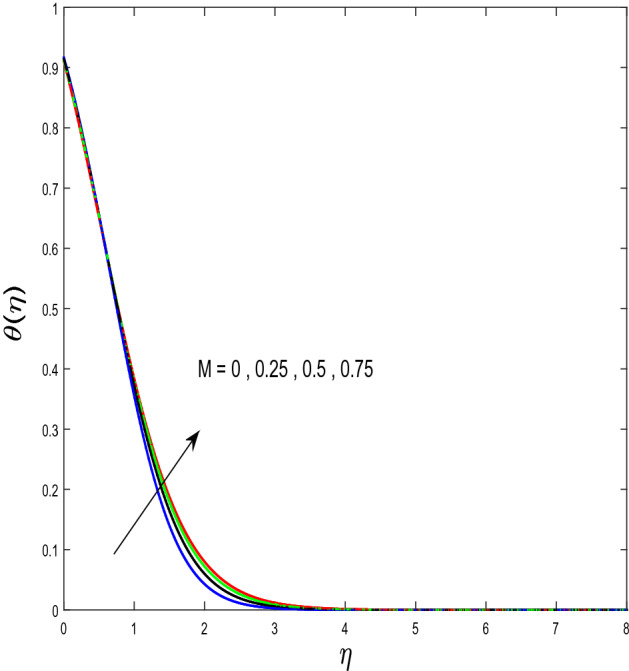
Plot of $\theta$ for $M$.

**Figure 9:**
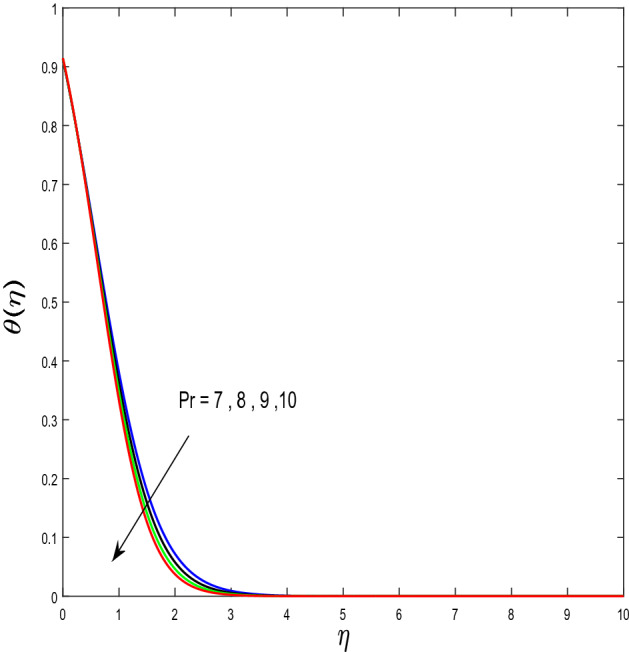
Plot of $\theta$ for $\Pr$.

**Figure 10:**
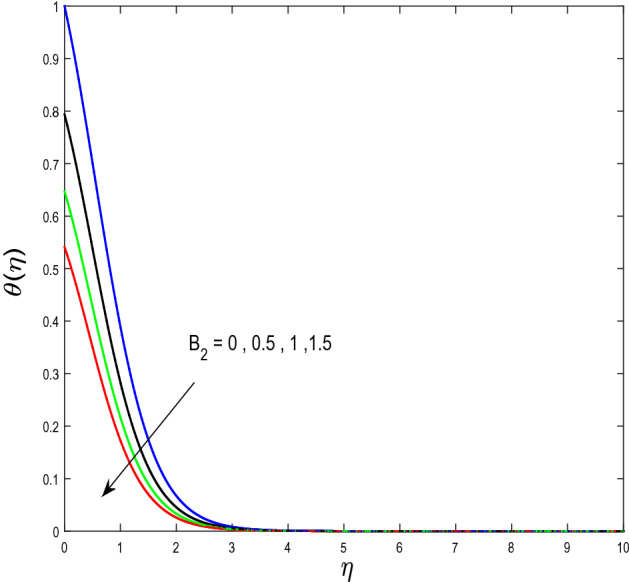
Plot of $\theta$ for $B_{2}$.

**Figure 11:**
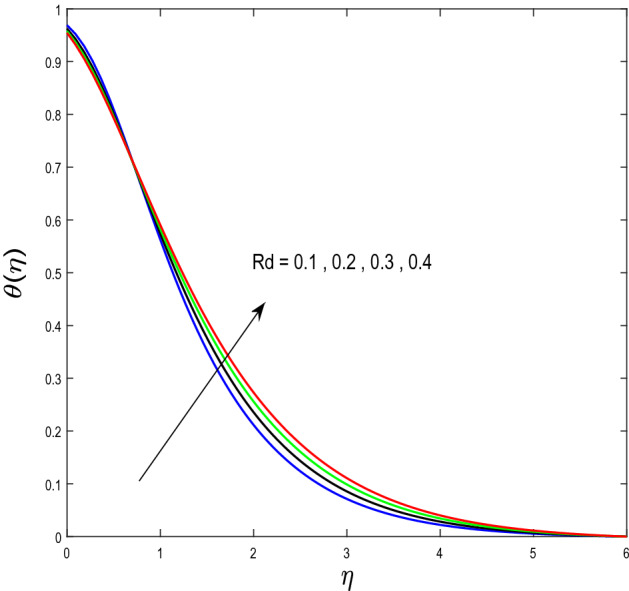
Plot of $\theta$ for $Rd$.

**Figure 12:**
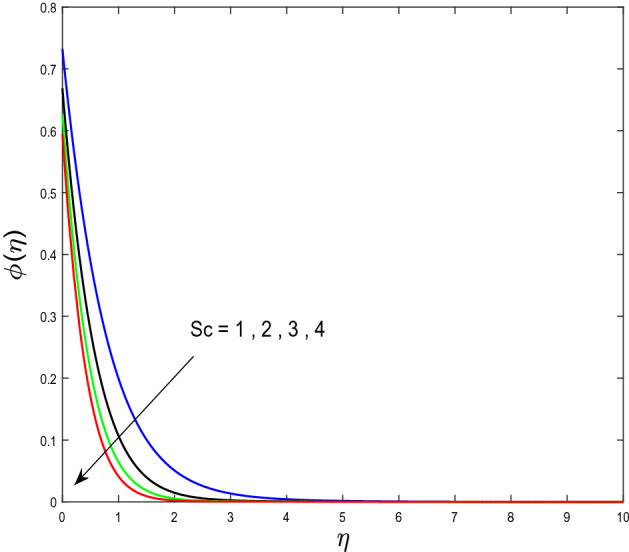
Plot of $\phi$ for $Sc$.

**Figure 13:**
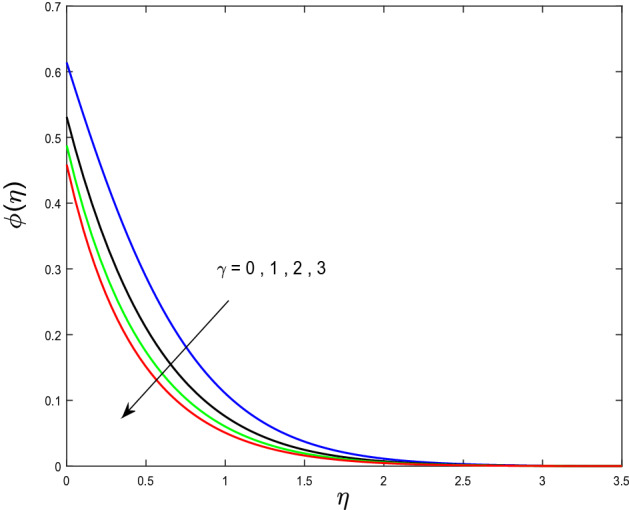
Plot of $\phi$ for $\gamma$.

**Figure 14:**
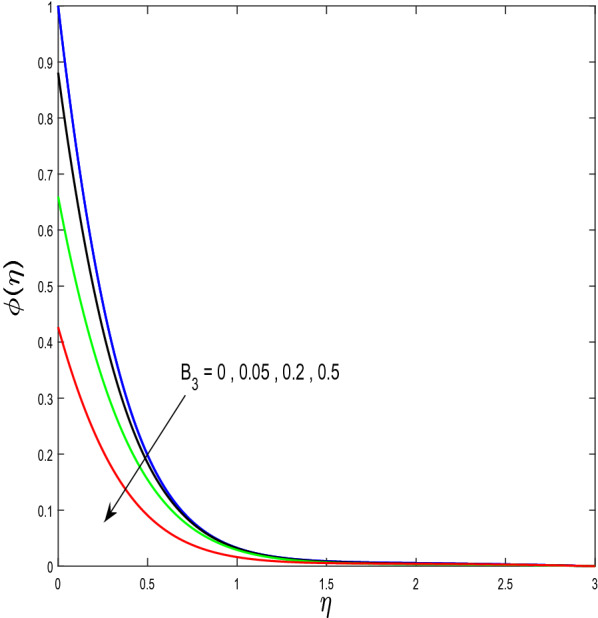
Behaviour of $\phi$ for $B_{3}$.

**Figure 15:**
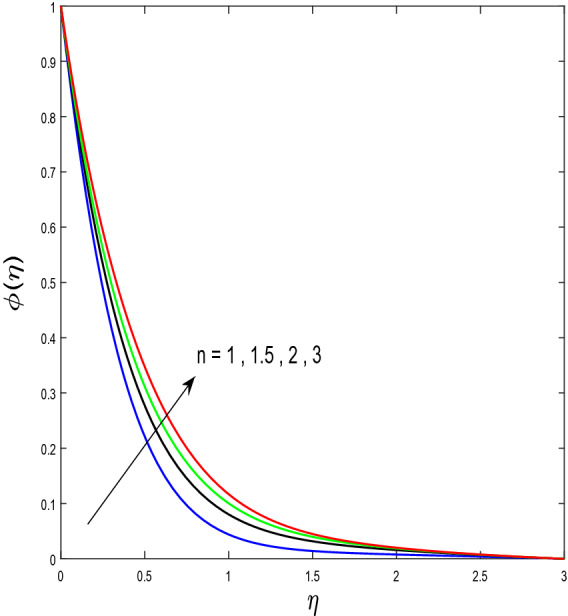
Plot of $\phi$ for $n$.

**Figure 16:**
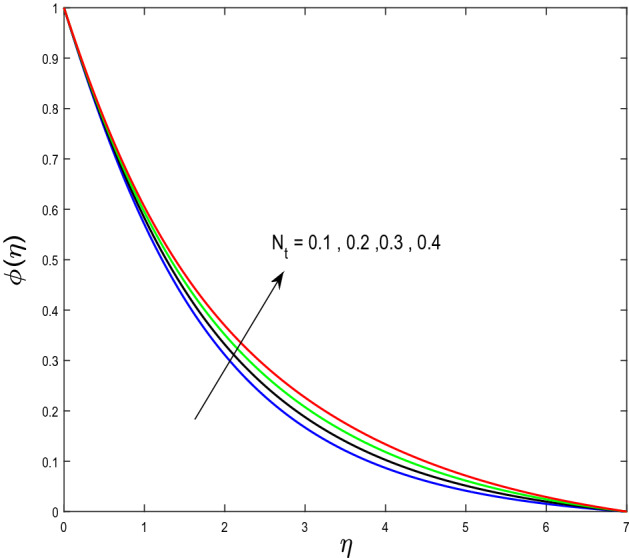
Plot of $\phi$ for $Nt$.

**Figure 17:**
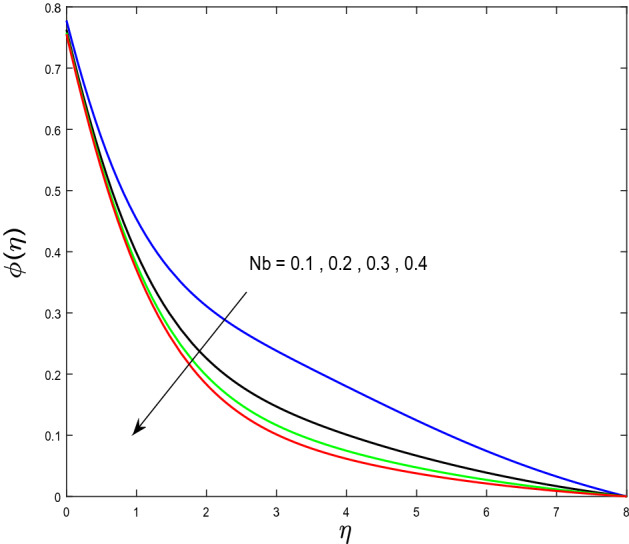
Plot of $\phi$ for $Nb$.

**Figure 18:**
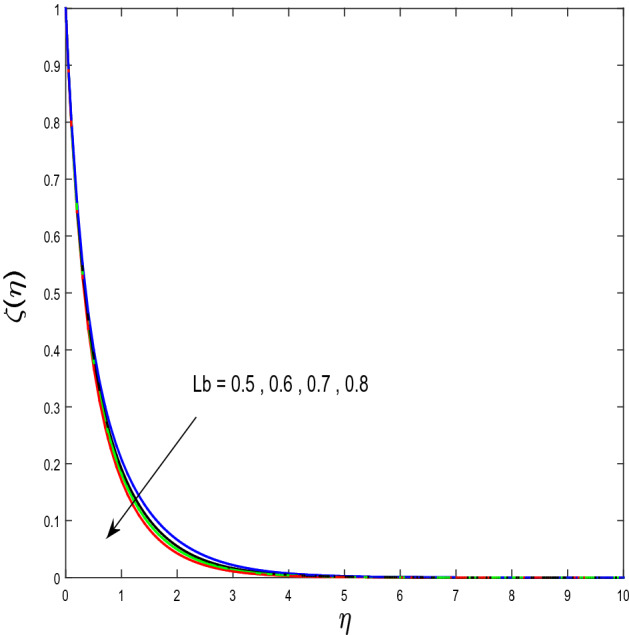
Plot of $\xi$ for $Lb$.

**Figure 19:**
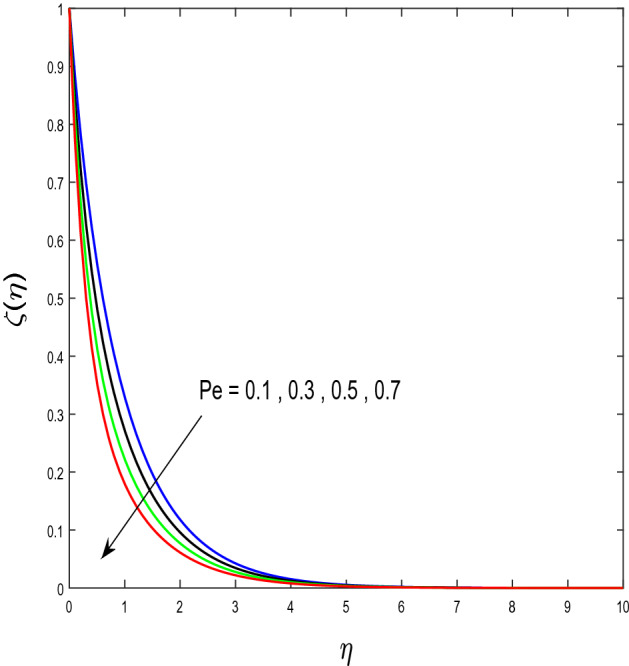
Plot of $\xi$ for $Pe$.

**Figure 20:**
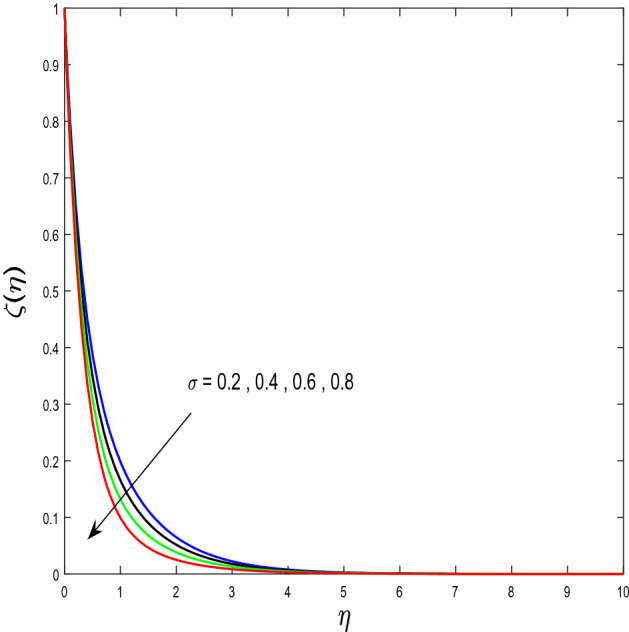
Plot of $\xi$ for $\sigma$.

**Figure 21:**
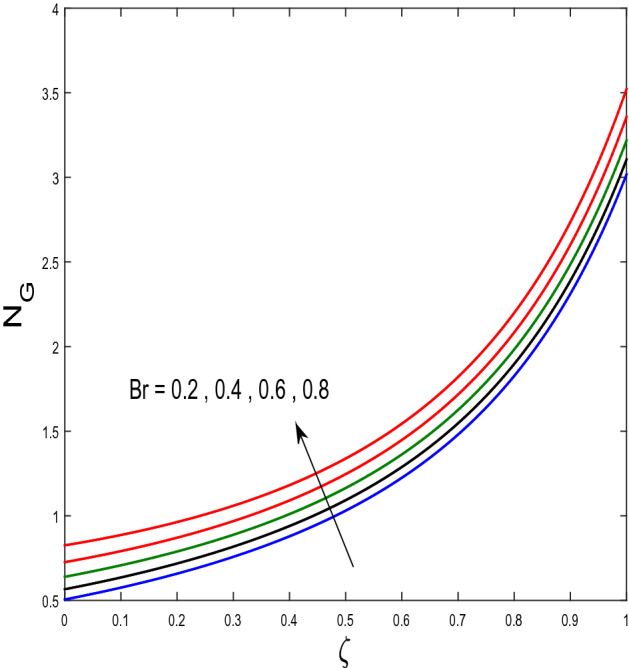
Plot of $N_{G}$ for $Br$.

**Figure 22:**
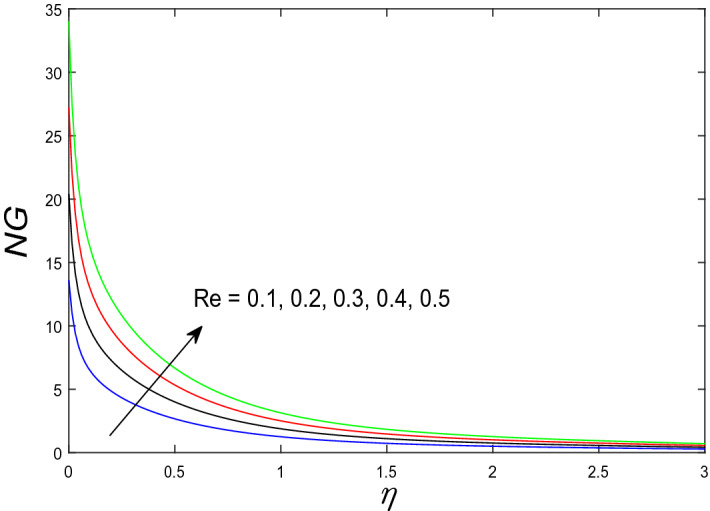
Plot of $N_{G}$ for ${\text{Re}}$.

In this segment, we will examine the effect of distinct parameters on velocity, concentration, temperature, and gyrotactic microorganism fields. The numerous parameters like the magnetic interaction parameter $(K),$ Darcy parameter $(Fr),$ radiation parameter $(Rd),$ Schmidt number $(Sc),$ temperature ratio parameter $(\theta_{w} ),$ porosity parameter $(\lambda ),$ Deborah number $(\beta ),$ thermophoresis parameter $(Nt),$ curvature parameter $(M),$ bioconvection Lewis number $(Lb),$ Prandtl number $(\Pr ),$ Brownian motion parameter $(Nb),$ wall roughness parameter $(B_{1} ),$ chemical reaction parameter $(\gamma ),$ concentration slip parameter $(B_{3} ),$ thermal slip parameter $(B_{3} ),$ Peclet number $(Pe),$ Bioconvection parameter $(\sigma ),$ and reaction order $n$ are discussed on temperature, velocity and nanoparticles concentration, and gyrotactic microorganism fields. Figure 2 demonstrated the behavior of $K$ on the $f^{^{\prime}} \left( \eta \right)$. The strength of the Lorentz force is measured by $K.$ The increase in $K$ enhances the Lorentz force strength and due to the rise in $K$ the velocity in axial direction decreases. As a result, the gradient of velocity at the surface is decreased. The wall $B_{1}$ effect on the velocity profile is described in Fig. 3. The slip decreases the speed close to the disk and this condition enhances by increasing in $K$. Practically, the stretched impact of a cylinder is moderately shifted to the liquid layers which result in a decrease in $f^{^{\prime}} \left( \eta \right)$. The influence of $Fr$ on the velocity field $f^{^{\prime}} \left( \eta \right)$ is investigated in Fig. 4. It is examined that by escalating the variations of $Fr$, the decreasing trend of the velocity field is seen. This is because the higher values of $Fr$ produce resistance in a liquid flow and hence velocity decreases. Figure 5 demonstrated the effect of the $\lambda$ on the velocity distribution of $f^{^{\prime}} \left( \eta \right)$. The liquid's velocity diminishes on greater estimations of the $\lambda$. Actually, the movement of the liquid is stalled because of the presence of permeable media, and this results in the falloff of the liquid velocity. The effect of $\beta$ on the velocity field $f^{^{\prime}} \left( \eta \right)$ is investigated in Fig. 6. It is examined that by escalating the variations of $\beta$, the diminishing behavior of the velocity field is seen. The impact of the $\theta_{w}$ upon $\theta \left( \eta \right)$ is explained by Fig. 7. A significant increase in $\theta \left( \eta \right)$ is observed. Enhancing $\theta_{w}$ signifies the temperature of the wall that causes thicker penetration depth for temperature profile. Likewise, the thermal diffusivity lies in the boundary layer with the relating exchange of heat. The thermal boundary layer corresponds to be larger nearby the region where the hotness is larger while it is lower a long way from cylinder because here temperature is low when compared to others. Subsequently, an intonation point emerges on the region when greater $\theta_{w}$ is considered. The temperature field for numerous $M$ is shown in Fig. 8. A generous upgrade in the temperature of the liquid is seen when the radius of the cylinder is reduced. The effect of $\Pr$ on the thermal profile is described in Fig. 9. It is observed that the existence of melting phenomenon of the liquid temperature increases with rising variations of $\Pr$. Therefore, we can judge that greater variation of $\Pr$ enhances the temperature field. Figure 10 defines that the large estimation of the $B_{2}$ descends the dimensionless liquid's temperature. From the figure, it is noticed that the thermal boundary layer becomes thicker on enhancing the values of the curvature parameter. Figure 11 is outlined to show the plots of $\theta \left( \eta \right)$ for numerous terms of $Rd$ when other variables are fixed. It can be judged that growing values of $Rd$ increase the temperature and its parallel thickness of layer become thicker. Figure 12 elucidates that an increment in $Sc$ decays the nanoparticle concentration distribution $\phi \left( \eta \right)$. There is an opposite relationship between the $Sc$ and the Brownian diffusion coefficient. Greater the values of Schmidt number $Sc$ lower will be the Brownian diffusion coefficient, which tends to decrease the $\phi \left( \eta \right)$. Figure 13 portrays the concentration field for different estimations of $\gamma$. Large variations of the $\gamma$ tend to smaller the nanoparticle concentration field. The descending behavior of a concentration profile $\phi \left( \eta \right)$ on a $B_{3}$ is drawn in Fig. 14. Figure 15 demonstrated that for greater values of reaction order $n$ the concentration profile becomes higher. Figure 16 depicts the influence of $Nt$ on $\phi \left( \eta \right)$. Both the concentration and thermal layer thickness are increased by accumulating the variations of the $Nt$. Greater estimations of the $Nt$ give rise to thermophoresis force which increases the movement of nanoparticles from cold to hot surfaces and also increases in the thermal layer thickness. The descending behavior in concentration distribution $\phi \left( \eta \right)$ against $Nb$ is shown in Fig. 17. An enhancement in $Nb$ increases the Brownian motion due to which there is an escalation in the movement of nanoparticles and hence boundary layer thickness reduces. Figure 18 plotted to draw the curves of $\xi \left( \eta \right)$ for different terms of $Lb$ while other variables are fixed. It is observed that $Lb$ depicts the decreasing behavior for large values of $Lb$. Figure 19 shows the behavior of $Pe$ on gyrotactic microorganisms’ profile $\xi \left( \eta \right)$. Here, $\xi \left( \eta \right)$ is an increasing function of $Pe$ that effects to a decrease the diffusivity of microorganisms. Figure 20 indicates the variations in gyrotactic microorganism profile $\xi \left( \eta \right)$ for distinct estimations of the$\sigma$. Large variations of $\sigma$ decrease the gyrotactic microorganism field. Figure 21 is drawn to illustrates the impact of the Brinkman numbers on the Entropy generation number. It is found that Entropy escalates for the Brinkman number. In Fig. 22, with rising estimations of ${\text{Re}}$, an increase is seen in the entropy generation number. A higher ${\text{Re}}$ causes more aggravation in the field and expand fluid friction and heat transfer, which eventually increases the rate of entropy in the boundary layer region.

Table 2 is generated to substantiate the outlined results in this study by comparing it with Khan and Mustafa^68^ and Tamoor et al.^56^ in limiting case. A good agreement between the two outcomes is seen. Besides, Tables 3, 4, 5 express the numerical variations of the local Sherwood number $Sh_{x}$, local Nusselt number $Nu_{x} ,$ and density amount of motile microorganism $Nn_{x}$ for distinct estimations of $K$, $M$, $\theta_{w}$, $Rd$, $\Pr$, $B_{1}$, $B_{2}$, $Sc$, $\gamma$, $k$, $Pe$, $Lb$, and $\sigma$. It is witnessed in Table 3 here that the heat flux rate is escalated for the growing estimates of $K$, but the opposite trend is perceived for the estimations of $M$, $\theta_{w}$, $Rd.$ In Table 4, it is witnessed that the mass flux rate is declined for the values of the $Sc$, and $\gamma$, however, it is enhanced for the increasing estimates of the $M$ and $n.$ The behavior of the varied parameters versus the density amount of motile microorganism is portrayed in Table 5. It is renowned that density amount of motile microorganism is improved for the estimations of $M$, $\Pr$, $\sigma$, $Pe$, and $Lb$.

**Table 2:** Validation of numerical outcomes for $- f^{\prime\prime}\left( 0 \right)$ with Khan and Mustafa^68^ and Tamoor et al.^56^ and when $M$ = $B_{1}$ = 0.

K	$- f^{\prime\prime}\left( 0 \right)$	$- f^{\prime\prime}\left( 0 \right)$	$- f^{\prime\prime}\left( 0 \right)$
	^68^	^56^	Present
0	1	1	1
0.2	1.0198039	1.01980	1.01981
0.5	1.1180340	1.11803	1.11803
0.8	1.2806248	1.28063	1.28062
1	1.4142136	1.41421	1.41421

**Table 3:** Computations of $\left( {{\text{Re}}_{x} } \right)^{\frac{1}{2}} Nu_{x}$ for various variations of $K$, $M$, $\theta_{w}$, $Rd$ when $\Pr$ = 7 and $B_{1}$ = $B_{2}$ = 0.5.

$K$	$M$	$\theta_{w}$	$Rd$	$\left( {{\text{Re}}_{x} } \right)^{{ - \frac{1}{2}}} Nu_{x}$
0.5	0.2	1.5		0.7654
1				0.6803
1.5				0.5851
2				0.5003
	0.5			0.7745
	0.7			0.7801
	1			0.7883
		2		1.1440
		2.5		1.4871
		3		1.7770
			0.1	1.3736
			0.3	2.2339
			0.5	3.3713

**Table 4:** Computations of $\left( {{\text{Re}}_{x} } \right)^{{ - \frac{1}{2}}} Sh_{x}$ for numerous variations of $Sc$,$M$, and $\gamma$ when $\Pr$ = 7, $k$ = 0.5 and $B_{1}$ = $B_{2}$ = $B_{3}$ = 0.5.

$M$	$Sc$	$\gamma$	$n$	$\left( {{\text{Re}}_{x} } \right)^{{ - \frac{1}{2}}} Sh_{x}$
0.2	5	1	1	1.15817
0.5				1.17480
0.7				1.18683
1				1.20568
	2			0.97565
	3			1.05478
	7			1.22726
		2		1.27036
		3		1.34142
		4		1.39264
			2	0.99641
			3	0.92496
			5	0.87854

**Table 5:** Computations of $\left( {{\text{Re}}_{x} } \right)^{{ - \frac{1}{2}}} Nn_{x}$ for various variations of $M$, $\Pr$, $\sigma$, $Pe$ and $Lb$.

$M$	$\Pr$	$\sigma$	$Pe$	$Lb$	$\left( {{\text{Re}}_{x} } \right)^{{ - \frac{1}{2}}} Nn_{x}$
0.3	1.5	0.2	0.3	0.2	0.935596
0.6					1.059580
0.9					1.193600
	1.0				0.915801
	1.5				0.935596
	2.0				0.940771
		0.2			0.935596
		0.4			1.012580
		0.6			1.089560
			0.4		1.065290
			0.5		1.189010
			0.6		1.307130
				0.3	0.963155
				0.6	1.038210
				0.9	1.103780

## Concluding remarks

In the current investigation, we have discussed nonlinear radiative MHD Williamson nano liquid flow via a stretched cylinder in a Darcy–Forchheimer porous media. The flow is assisted by the impacts of the chemical reaction, and gyrotactic microorganisms with partial slip condition at the boundary. The solution to the problem is addressed by the MATLAB scheme of the bvp4c built-in function. The main results of the present investigation are appended below:

- $Nb$ and $Nt$ show the opposing nature against the concentration field.
- The velocity distribution is lowered for large variations of $K$, $Fr,$ and $\lambda .$
- $Pe$ decreases the gyrotactic microorganism profile.
- An increment in $$Sc,$$$$B_{3}$$, and $$\gamma$$ leads to a lowering concentration profile.
- Enhanced variations of $\Pr$ and $B_{2}$ display the decreasing behavior on the temperature profile.
- Large values of $\theta_{w}$, $Rd,$ and $M$ causes an increment in temperature distribution.
- Gyrotactic microorganism profile reduces for greater variations of $Lb$ and $\sigma$.
- Entropy intensifies for $Br$; however, an opposite tendency is observed for $\beta$.

## List of symbols

$B_{0}$: Magnetic field intensity
$B_{1}$: Wall roughness parameter
$B_{2}$: Wall thermal parameter
$B_{3}$: Concentration slip parameter
$B_{4}$: Motile slip parameter
$$Br$$: Brinkman number
$D_{T}$: Thermophoretic diffusion coefficient (${\text{m}}^{2} /{\text{s}}$)
$$c_{b}$$: Capacity
$C_{{f_{x} }}$: Drag force coefficients
$k_{2}$: Permeability of porous medium
$$C_{w}$$: Wall’s concentration
$D_{B}$: Brownian diffusion coefficient (${\text{m}}^{2} /{\text{s}}$)
$C_{\infty }$: Ambient liquid concentration
$$C$$: Liquid concentration ($${\text{kg}}\,{\text{m}}^{ - 3}$$)
$c_{p}$: Heat Specific capacity (${\text{J}}\,{\text{kg}}^{ - 1} \,\,{\text{k}}^{ - 1}$)
$D_{n}$: Diffusivity of the microorganism (${\text{m}}^{2} /{\text{s}}$)
$$Pe$$: Peclet numbers
$F$: Non-uniform inertial coefficient
$$Fr$$: Forchheimer number
$L_{4}$: Motile slip coefficient
$$l$$: Characteristic length $$\left( {\text{m}} \right)$$
$$j_{w}$$: Mass flux ($${\text{m}}/{\text{s}}$$)
$Lb$: Bioconvection Lewis number
$K$: Magnetic interaction parameter
$L_{2}$: Thermal slip coefficient
$$k^{*}$$: Mean absorption coefficient $$\left( {{\raise0.7ex\hbox{$1$} \!\mathord{\left/ {\vphantom {1 {\text{m}}}}\right.\kern-\nulldelimiterspace} \!\lower0.7ex\hbox{${\text{m}}$}}} \right)$$
$q_{n}$: Surface motile microorganisms (${\text{W}}/{\text{m}}^{2} {\text{K}}$)
$Nb$: Brownian motion parameters
$$u_{0}$$: Reference velocity ($${\text{m}}/{\text{s}}$$)
$$n$$: Reaction order
$$k$$: Thermal conductivity $$\left( {{\raise0.7ex\hbox{${\text{W}}$} \!\mathord{\left/ {\vphantom {{\text{W}} {\text{m}}}}\right.\kern-\nulldelimiterspace} \!\lower0.7ex\hbox{${\text{m}}$}}} \right)$$
$L_{1}$: Velocity slip coefficient
$N_{G}$: Rate of Entropy generation
$L_{3}$: Concentration slip coefficient
$Nu_{x}$: Local Nusselt number
$$M$$: Curvature parameter $$\left( {{\text{m}}^{ - 1} } \right)$$
$$q_{w}$$: Wall heats ($${\text{W}}/{\text{m}}^{2}$$)
$$Nt$$: Thermophoresis parameter
$$\Pr$$: Prandtl number
$q_{r}$: Radiation heat flux (${\text{W}}/{\text{m}}^{2}$)
$Nn_{x}$: Density number of motile microorganisms
$$r$$: Radial coordinate $$\left( m \right)$$
$$Rd$$: Radiation parameter
$$T_{w}$$: Wall’s temperature ($${\text{K}}$$)
$$\sigma_{1}$$: Electrical conductivity $$\left( {{\raise0.7ex\hbox{${\text{S}}$} \!\mathord{\left/ {\vphantom {{\text{S}} {\text{m}}}}\right.\kern-\nulldelimiterspace} \!\lower0.7ex\hbox{${\text{m}}$}}} \right)$$
$u$: Velocity component along $x$-direction (${\text{m}}/{\text{s}}$)
$$Sc$$: Schmidt number
$$L$$: Diffusion parameter
$T_{\infty }$: Ambient fluid temperature (${\text{K}}$)
$$\sigma^{*}$$: Stefan–Boltzmann constant $$\left( {{\raise0.7ex\hbox{${\text{W}}$} \!\mathord{\left/ {\vphantom {{\text{W}} {{\text{m}}^{2} {\text{K}}^{4} }}}\right.\kern-\nulldelimiterspace} \!\lower0.7ex\hbox{${{\text{m}}^{2} {\text{K}}^{4} }$}}} \right)$$
$w_{c}$: Constant all-out cell swimming speed (${\text{m}}/{\text{s}}$)
$$S_{G}$$: Entropy generation
$$\sigma$$: Bioconvection parameter
$$\Delta T$$: Temperature difference
$Sh_{x}$: Local Sherwood number
$v$: Velocity component along $r$-direction (${\text{m}}/{\text{s}}$)
$$x$$: Axial coordinate $$\left( {\text{m}} \right)$$
$$u_{w}$$: Stretching velocity ($${\text{m}}/{\text{s}}$$)

## Greek symbols

$$\alpha$$: Thermal diffusivity ($${\text{m}}^{2} /{\text{s}}$$)
$\alpha_{1}$: Temperature difference parameter
$\alpha_{2}$: Concentration difference parameter
$\alpha_{3}$: Motile difference parameter
$\beta$: Deborah number in terms of velocity time
$\gamma$: Chemical reaction parameter
$$\lambda$$: Porosity parameter
$$\lambda_{1}$$: Reaction rate
$$\lambda^{*}$$: Relaxation time
$$\nu$$: Kinematic viscosity ($${\text{m}}^{2} /{\text{s}}$$)
$$\rho$$: Density ($${\text{kg}}/{\text{m}}^{3}$$)
$$\mu$$: Absolute viscosity ($${\text{Pa}}/{\text{s}}$$)
$$\eta$$: Similarity variable
$$\psi$$: Stream function ($${\text{m}}^{2} /{\text{s}}$$)
$\theta_{w}$: Temperature ratio parameter
$\tau$: The quotient of the effective heat capacity of the nano-particle material and of the fluid

## Subscripts

$\infty$: Condition at the infinity
$w$: Condition at the wall
